# The Role of Water in Lanthanide-Catalyzed Carbon–Carbon Bond Formation

**DOI:** 10.3390/molecules17022073

**Published:** 2012-02-20

**Authors:** Derek J. Averill, Prabani Dissanayake, Matthew J. Allen

**Affiliations:** Department of Chemistry, Wayne State University, Detroit, MI 48202, USA

**Keywords:** luminescence decay, aqueous catalysis, lanthanide

## Abstract

Luminescence-decay measurements in combination with high-performance liquid chromatography analyses were used to study the relationship between rates of catalysis and water-coordination numbers of europium-based precatalysts in the aqueous Mukaiyama aldol reaction. A correlation between reactivity and water-coordination number was observed and is reported here.

## 1. Introduction

**Figure 1:**
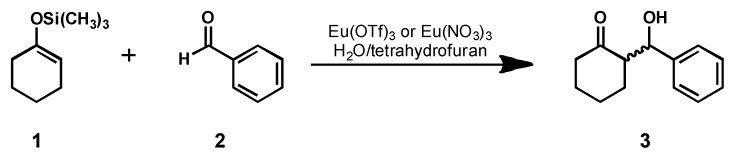
The water-tolerant Mukaiyama aldol reaction between silyl enol ether **1**, and benzaldehyde (**2**), studied in this work.

Some of the most important transformations in organic chemistry result in the formation of carbon–carbon and carbon–heteroatom bonds, and both of these bonds can be formed using lanthanide triflate [Ln(OTf)_3_]-containing precatalysts [1,2,3,4,5,6,7,8,9,10,11,12,13,14]. Lanthanide triflates are reusable, easy-to-handle, and can function as strong Lewis acids in both aqueous and non-aqueous solvent mixtures [1]. Water-tolerant Lewis acid catalysts are advantageous relative to water-sensitive catalysts because water-tolerant Lewis acids eliminate the need to rigorously dry solvents before use. Consequently, the Lewis-acidic and water-tolerant features of lanthanide(III) salts have aroused much interest in aqueous lanthanide-catalyzed bond-forming reactions [1,2,3,4,5,6]. We are studying the Mukaiyama aldol reaction between a silyl enol ether and an aldehyde because this reaction can be both water-tolerant and stereoselective, making it an important carbon–carbon bond-forming reaction (Figure 1) [3,4,15,16].

We hypothesized that the water-coordination numbers of lanthanide-based precatalysts influenced the reaction rate and final yield of this reaction. In aqueous solution, lanthanide(III) ions have relatively fast inner-sphere water-exchange rates (~10^8^ s^–1^) [17], and each site of exchanging water represents a potential site for benzaldehyde coordination. We hypothesized that a larger water-coordination number would result in greater probability for aldehyde coordination and, consequently, a faster reaction rate because bound aldehyde, **2**, is activated for nucleophilic attack by enol ether, **1** (Figure 1). 

We have previously reported the use of luminescence-decay measurements to monitor the average water-coordination number of Eu(OTf)_3_ in mixtures of water with organic co-solvents in the presence and absence of substrates [18,19]. In this article, we contribute to the mechanistic understanding of the aqueous lanthanide-catalyzed Mukaiyama aldol reaction by correlating the water-coordination numbers of europium-based precatalysts with steady state reaction rates. Further, we describe the influence of europium counteranions on reaction yields and steady state reaction rates.

## 2. Results and Discussion

**Figure 2:**
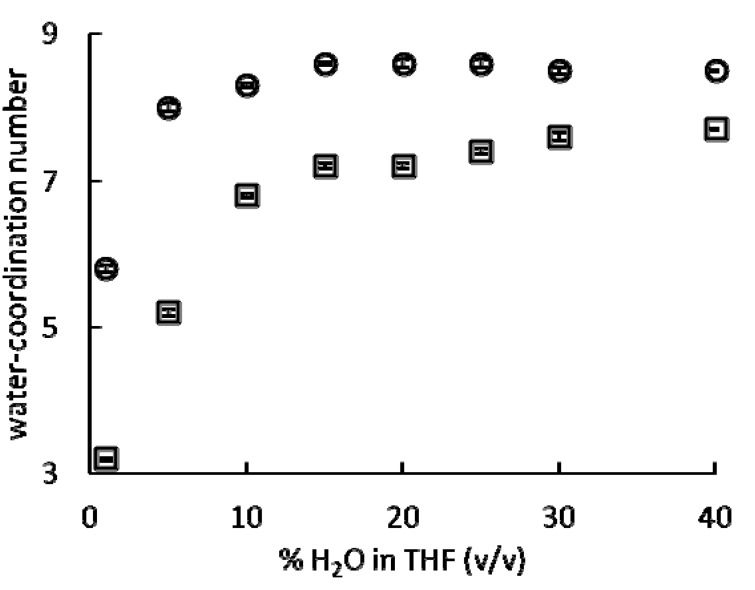
Water-coordination numbers as a function of solvent ratio for Eu(OTf)_3_ (○) and Eu(NO_3_)_3_ (□) in mixtures of H_2_O and THF. Experiments were performed with 7 mol % precatalyst. Water-coordination numbers of Eu(OTf)_3_ (○) in 5, 10, 20, 30, and 40% H_2_O in THF (v/v) are from reference [19]. Error bars represent the standard error of the mean of between three and nine independent measurements.

Water-coordination numbers were determined using measured luminescence-decay rates with Equation 1, where 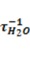 and 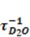 represent the measured decay rates in H_2_O and D_2_O, respectively; *q* represents the average water-coordination number; and α accounts for the influence of non-coordinated molecules on luminescence decay [19]. We found the average water-coordination numbers of the studied europium salts in H_2_O/THF mixtures ranging from 1 to 40% H_2_O in THF (v/v) to be between 3.2 and 8.6 water molecules (Figure 2, Table 1).

These values are in agreement with previous lanthanide-coordination studies which show a maximum coordination number between 8 and 9 [18,19,20]. 


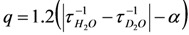
(1)


**Figure 3:**
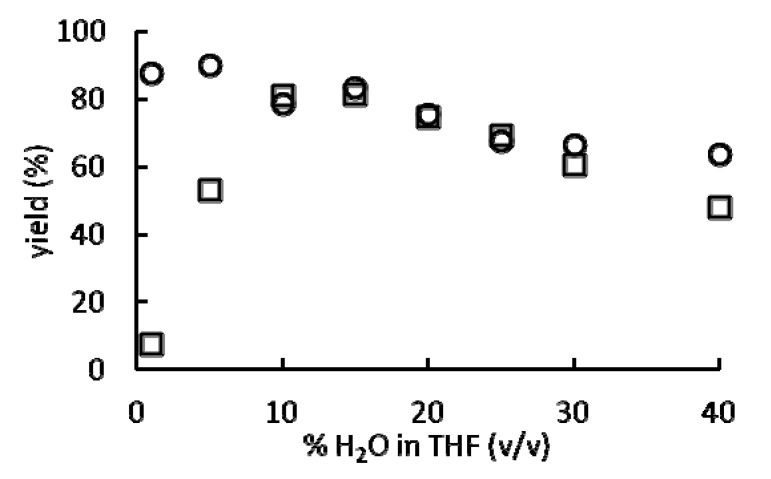
Yields of the reaction shown in Figure 1 catalyzed by Eu(OTf)_3_ or Eu(NO_3_)_3_ after 48 h as a function of solvent composition. Conditions: solvent mixtures of 1 to 40% H_2_O in THF (v/v) containing 7 mol % Eu(OTf)_3_ (○) or Eu(NO_3_)_3_ (□).

We hypothesized that Eu(NO_3_)_3_ should have lower catalytic activity than Eu(OTf)_3_, an effective Lewis acid precatalyst, because of its lower water-coordination numbers. By studying Eu(OTf)_3_ and Eu(NO_3_)_3_, we were able to assess the effects of counteranions on the catalytic activity of europium. We chose to use these precatalysts because the water-coordination numbers of europium ions in aqueous solutions are influenced by the composition of the solvent and the identity of the counteranions (Figure 2). Due to the limited water solubility of **1**, we were unable to study **1** at water percentages of less than 40% H_2_O in THF (v/v). To test our hypothesis that water-coordination numbers influence the steady state reaction rate and final yield of this reaction, the yields of Eu(OTf)_3_- and Eu(NO_3_)_3_-catalyzed Mukaiyama aldol reactions were measured after 48 h in solvent mixtures ranging from 1 to 40% H_2_O in THF (v/v) (Figure 3). Yields were measured after 48 h because Eu(NO_3_)_3_-catalyzed reactions in 1, 5, and 10% H_2_O in THF (v/v) required longer than 24 h to reach completion. As shown in Figure 3, Eu(OTf)_3_- and Eu(NO_3_)_3_-catalyzed Mukaiyama aldol reactions afforded the highest yields at 5 and 15% H_2_O in THF (v/v), respectively. Interestingly, 5 and 15% H_2_O in THF (v/v) roughly correspond to the solvent composition at which increasing the H_2_O concentration has the least effect on the water-coordination number for both Eu(OTf)_3_ and Eu(NO_3_)_3_ (Figure 2).

**Table 1:** Mean water-coordination numbers (*q*) of (a) Eu(OTf)_3_ and (b) Eu(NO_3_)_3_ in mixtures of H_2_O/THF. Error represents standard error of the mean of between 3 and 9 measurements.

(a)			(b)	
% H_2_O in THF (v/v)	*q*		% H_2_O in THF (v/v)	*q*
1	5.8 ± 0.1		1	3.2 ± 0.02
5	8.0 ± 0.1 *		5	5.2 ± 0.04
10	8.3 ± 0.03 *		10	6.8 ± 0.03
15	8.6 ± 0.02		15	7.2 ± 0.03
20	8.6 ± 0.1 *		20	7.2 ± 0.04
25	8.6 ± 0.1		25	7.4 ± 0.04
30	8.5 ± 0.04 *		30	7.6 ± 0.1
40	8.5 ± 0.01 *		40	7.7 ± 0.01

nd = not determined, * from reference [19].

**Figure 4:**
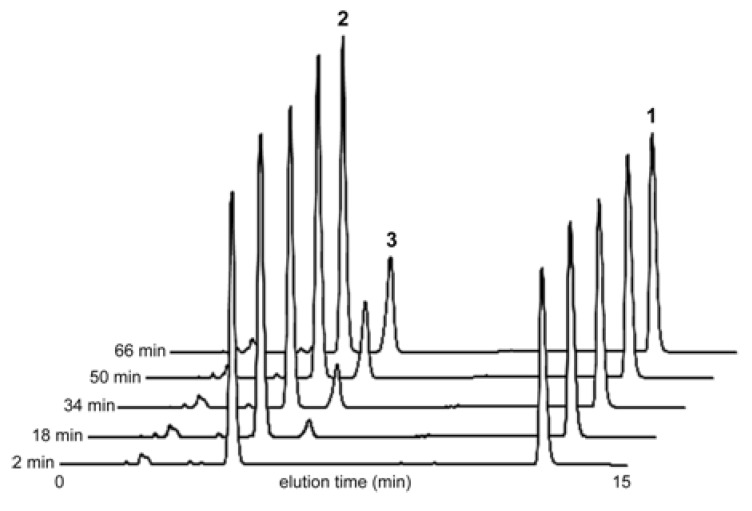
HPLC traces of 7 mol % Eu(OTf)_3_-catalyzed Mukaiyama aldol reaction in 10% H_2_O/THF (v/v) after 2, 18, 34, 50, and 66 min. The increase in peak 3 with time corresponds to an increase in product concentration. The y-axis shows absorbance at 210 nm in arbitrary units.

We also investigated the relationship between the water-coordination numbers of europium precatalysts and the steady state reaction rates of the Mukaiyama aldol reaction shown in Figure 1. For these studies, Eu(OTf)_3_ or Eu(NO_3_)_3_ was used in solvent mixtures ranging from 1 to 40% H_2_O in THF (v/v) and Eu(OTf)_3_ in THF. These europium-containing precatalysts and solvent mixtures were used because of the wide range of water-coordination numbers (3.2 to 8.6) accessible under these conditions (Figure 2). We expected that this range of water-coordination numbers would allow us to observe changes in reactivity to test our hypothesis regarding the relationship between steady state reaction rate and water-coordination number. To determine the steady state reaction rates of Eu(NO_3_)_3_- and Eu(OTf)_3_-catalyzed Mukaiyama aldol reactions, we monitored the concentration of product, **3**, at 2, 18, 34, 50, and 66 min using HPLC (Figure 4). 

**Figure 5:**
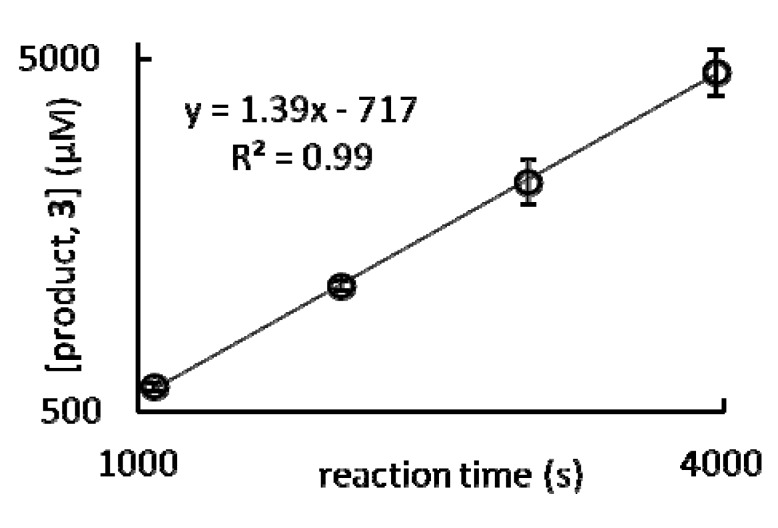
Plot of the formation of product, 3, as a function of time. Conditions: 7 mol % Eu(OTf)_3_ in 10% H_2_O/THF (v/v) after 1080, 2040, 3000, and 3960 s. The steady state reaction rate derived from this plot is the slope of the best fit line and is 1.39 μM s^–1^. Error bars represent the standard error of the mean of three independently prepared samples.

From these HPLC traces and a calibration curve for **3** produced using the same conditions, the area under the peaks was used to determine concentration. An example of the resulting data is plotted in Figure 5, which exemplifies the linear increase of product concentration as a function of time for the conditions studied between 18 and 66 min. By monitoring the linear increase in product, **3**, concentration as a function of time we were able to calculate the steady state reaction rates as the slope of the best fit line in Figure 5 [21]. Table 2 contains a complete list of steady state reaction rates.

**Figure 6:**
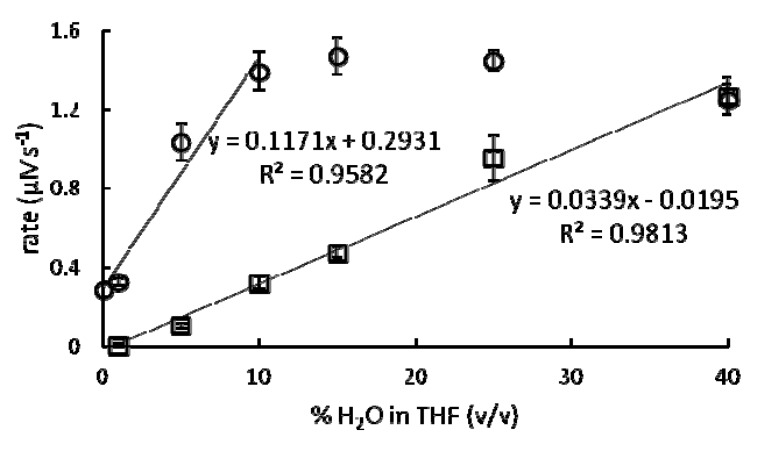
Steady state reaction rates of 7 mol % Eu(OTf)_3_- (○) or Eu(NO_3_)_3_-catalyzed (□) Mukaiyama aldol reactions in 0–40% H_2_O/THF mixtures (v/v). Regression lines represent the rate dependence on anion identity and solvent composition. Note: 10% H_2_O in THF (v/v) corresponds to the solvent composition at which Eu(OTf)_3_ is saturated with water (Figure 2). Error bars represent the standard error of the mean of three independently prepared samples.

From the data in Figure 5 and data from similar experiments using Eu(OTf)_3_ or Eu(NO_3_)_3_ in a range of solvents [0–40% H2O in THF (v/v)], a relationship was observed between the steady state reaction rates of europium-catalyzed Mukaiyama aldol reactions and solvent composition (Figure 6). Reactions catalyzed by Eu(OTf)_3_ had faster steady state reaction rates than reactions catalyzed by Eu(NO_3_)_3_ in every solvent composition studied. This observation can be rationalized based upon relative binding affinities of the anions for Eu^3+^, which affect the water-coordination numbers of the precatalysts: triflate has a lower binding affinity for lanthanide(III) ions than nitrate [22].

**Table 2:** Mean steady state reaction rates of 7 mol % Eu(OTf)_3_ (a) or 7 mol % Eu(NO_3_)_3_ (b) catalyzed Mukaiyama aldol reactions. Error represents standard error of the mean of 3 independent samples.

(a)			(b)	
% H_2_O in THF (v/v)	steady state reaction rate (μM s^–1^)		% H_2_O in THF (v/v)	steady state reaction rate (μM s^–1^)
0	0.29 ± 0.06		0	nd
1	0.33 ± 0.03		1	0.008 ± 0.001
5	1.0 ± 0.2		5	0.11 ± 0.02
10	1.4 ± 0.2		10	0.33 ± 0.06
15	1.5 ± 0.2		15	0.48 ± 0.04
20	nd		20	nd
25	1.45 ± 0.08		25	1.0 ± 0.2
30	nd		30	nd
40	1.3 ± 0.1		40	1.3 ± 0.2

nd = not determined.

This difference in europium binding affinities between triflate and nitrate results in higher water-coordination numbers for Eu(OTf)_3_ compared to Eu(NO_3_)_3_ and, ultimately, corresponds to higher europium accessibility because each water molecule coordinated to Eu^3+^ represents a potential site for benzaldehyde coordination and activation for reaction. In general, increasing water percentages resulted in faster steady state reaction rates, but the steady state reaction rates of Eu(OTf)_3_-catalyzed reactions reached a maximum and remained constant at solvent mixtures containing greater than 10% H_2_O in THF (v/v) (Figure 6). 

This solvent composition [10% H2O in THF (v/v)] corresponds to the lowest H_2_O concentration at which the water-coordination number is at a maximum value (Figure 2). The increase of steady state reaction rates of Eu(NO_3_)_3_-catalyzed reactions over the entire range of solvents in this study can be attributed to the water-coordination number of Eu(NO_3_)_3_ that increases without reaching a maximum with increasing H_2_O in THF from 1 to 40%. An alternative explanation for our observations with Eu(NO_3_)_3_ is the determination of rate by a dynamic involvement of NO_3_^–^ in the inner-sphere of the lanthanide ion. However, the slower steady state reaction rates of Eu(NO_3_)_3_-catalyzed Mukaiyama aldol reactions relative to Eu(OTf)_3_-catalyzed reactions support our hypothesis that larger water-coordination numbers and less strongly binding counteranions enable faster reaction rates.

## 3. Experimental Section

### 3.1. General

Unless otherwise noted, purchased chemicals were used as supplied. Tetrahydrofuran (THF) was purified using a solvent purification system (Vacuum Atmospheres Company), and water was purified using a PURELAB Ultra Mk2 (ELGA) water purification system. 2-(Hydroxyphenylmethyl)- cyclohexanone (**3**), was synthesized according to a published procedure [1]. Flash chromatography was performed using silica gel 60, 230–400 mesh (EMD Chemicals). Thin layer chromatography (TLC) was performed on silica gel 60 coated ASTM TLC plates F_254_ (250 μm thickness). TLC visualization was accomplished using a hand-held UV lamp followed by staining with potassium permanganate (2 g KMnO_4_, 20 g K_2_CO_3_, 5 mL 5% w/v aqueous NaOH, 300 mL H_2_O). High-performance liquid chromatography (HPLC) analyses were performed on a Shimadzu HPLC system equipped with a C18 column (Zorbax Eclipse XDB-C18, 3.5 μm, 4.6 × 150 mm). Detection of eluent was carried out with a photodiode array detector at 210 nm. HPLC analyses used a binary gradient method (pump A: water, pump B: acetonitrile; 40–90% B over 15 min; flow rate: 1 mL/min). Europium concentrations were verified using xylenol orange according to a published procedure [23].

### 3.2. Mukaiyama Aldol Reaction Protocol

Mukaiyama aldol reactions were carried out at ambient temperature in 0, 1, 5, 10, 15, 20, 25, 30, or 40% H_2_O in THF (v/v) (3.0 mL) containing either Eu(OTf)_3_ or Eu(NO_3_)_3_ (1.2 mM); to each solution, **1** (20.0 μL, 0.100 mmol) and **2** (5.0 μL, 0.050 mmol) were added using gas tight syringes. Immediately after preparation, each reaction mixture was vigorously shaken for 10 s and passed through a 0.20 μm syringe filter into an HPLC autosampler vial. Analyses were performed to determine product concentration using HPLC. Compounds **1**, **2**, and **3** eluted in the order **2**, **3**, and **1**. For the quantitative determination of products, a calibration curve of **3** from 0.0 to 4.0 mg/mL in 1:1 H_2_O/THF (v/v) was made from peak integrations using the same HPLC conditions.

### 3.3. Luminescence-Decay Measurements

Water-coordination numbers were determined by acquiring luminescence-decay measurements using a HORIBA Jobin Yvon Fluoromax-4 spectrofluorometer in decay-by-delay scan mode using the phosphorescence lifetime setting. Experimental details and data analyses were performed according to previously described methods [19].

## 4. Conclusions

We have reported the dynamic luminescence-decay measurements of Eu(NO_3_)_3_ and Eu(OTf)_3_ in binary solvent mixtures. In addition to monitoring the water-coordination numbers of these europium-containing precatalysts, we measured the yields and steady state reaction rates of the Mukaiyama aldol reaction catalyzed by these salts in solvent mixtures from 1 to 40% H_2_O in THF (v/v), and the steady state rate of the Eu(OTf)_3_-catalyzed Mukaiyama aldol in THF. From these measurements, we found a correlation between steady state reaction rate and water-coordination number as well as between yield and solvent composition. The use of luminescence-decay measurements to probe the coordination environment of europium-based precatalysts in solution enabled the study of the influence of precatalyst coordination-environment on steady state reaction rate. These results are useful in the design of new precatalysts to be used for aqueous, enantioselective, lanthanide-catalyzed bond forming reactions because they suggest that faster rates of catalysis will require lower ligand coordination numbers. Further, the methodology described here can be applied to other lanthanide-catalyzed bond-forming reactions in aqueous media to gain a better understanding of the influence of water on the structure–activity relationship between precatalysts and rates of catalysis.
